# Christopher Heath, F.R.C.S

**Published:** 1905-10

**Authors:** 


Christopher Heath, F.R.C.S, died suddenly at his home in London, August 8, 1905, aged 70 years. Mr. Heath was one of the most prominent English surgeons, and a teacher and author of world-wide fame.

